# Cognitive and Neuroimaging Divergence Between Juvenile and Adult FUS Amyotrophic Lateral Sclerosis

**DOI:** 10.1002/acn3.70447

**Published:** 2026-06-15

**Authors:** Alexandra V. Jürs, Marcel Naumann, Annaliis Lehto, Hanna Schön, Jens Kurth, Johannes Prudlo, Andreas Hermann, Elisabeth Kasper

**Affiliations:** ^1^ Translational Neurodegeneration Section “Albrecht Kossel”, Department of Neurology University Medical Center Rostock Rostock Germany; ^2^ German Center for Neurodegenerative Diseases (DZNE) Rostock/Greifswald Rostock Germany; ^3^ Institute of Diagnostic and Interventional Radiology, Pediatric Radiology and Neuroradiology University Medical Center Rostock Rostock Germany; ^4^ Department of Nuclear Medicine Medical Center Rostock Rostock Germany; ^5^ Department of Neurology University Medical Center Rostock Rostock Germany; ^6^ Center for Transdisciplinary Neuroscience Rostock (CTNR) University Medical Center Rostock Rostock Germany

**Keywords:** amyotrophic lateral sclerosis, cognitive function, FUS, intellectual disability, juvenile‐onset ALS, neuroimaging

## Abstract

**Objective:**

Amyotrophic lateral sclerosis (ALS) is a neurodegenerative disorder characterized by progressive motor neuron degeneration. *Fused in sarcoma* (FUS)‐associated juvenile ALS (jALS) represents a distinct and aggressive subgroup with rapid deterioration and poor prognosis. Certain *FUS* mutations have been associated with comorbid intellectual disability, suggesting neurodevelopmental involvement. We compared FUS‐jALS with adult‐onset FUS‐ALS cases (aALS) to evaluate the association between premorbid cognitive impairment, genetic and clinical features incorporating neuroimaging data.

**Methods:**

Patients with genetically confirmed FUS‐ALS were classified as jALS (onset < 25 years) or aALS (onset ≥ 25 years). Neuropsychological assessment used Mehrfachwahl‐Wortschatz‐Test (MWT) for verbal IQ, and the Edinburgh Cognitive and Behavioral ALS Screen (ECAS), with cognitive impairment classified according to Strong criteria. Volumetric analysis was conducted on structural MRI and FDG‐PET data.

**Results:**

All three jALS (P525L [*n* = 2], H517_Q519del [*n* = 1]) showed rapid progression with early severe clinical events. Neuropsychological assessment revealed global cognitive deficits (ALS‐ci) with widespread dysfunction beyond typical ALS‐specific patterns and reduced verbal IQ, pointing towards premorbid cognitive impairment. aALS demonstrated slower progression and were predominantly cognitively unimpaired (ALS‐ni) or showed an ALS‐specific impairment. Neuroimaging revealed distinct patterns: jALS cases demonstrated posterior cortical atrophy and hypometabolism on FDG‐PET, while aALS showed largely preserved brain volumes and limbic‐subcortical hypometabolism.

**Interpretation:**

Specific *FUS* mutations (P525L, H517_Q519del) predispose to jALS with severe progression and premorbid cognitive impairments, supporting a genotype–phenotype association. Posterior cortical findings suggest neurodevelopmental delay rather than disease‐related neurodegeneration. Genetic *FUS* screening may be warranted in patients with intellectual disability and motor signs, given emerging targeted therapies.

## 1 Introduction

Amyotrophic lateral sclerosis (ALS) is a heterogeneous neurodegenerative disorder primarily affecting upper and lower motor neurons, typically leading to death within 3–5 years after diagnosis [1, 2]. Up to 50% of patients develop non‐motor manifestations, including cognitive impairment and behavioral abnormalities [3]. ALS‐specific cognitive impairments comprise, in particular, deficits in executive functions, language abilities, and social cognition [4].

While approximately 90% of cases are classified as sporadic ALS (sALS), around 10% represent familial ALS (fALS), typically inherited in an autosomal‐dominant pattern [5]. To date, more than 40 ALS‐associated genes have been identified, accounting for 50%–85% of fALS cases and 10%–20% of sALS cases [6, 7]. Pathogenic variants in the *Fused in Sarcoma* (*FUS*) gene account for approximately 1% of sALS and up to 5% of fALS cases in European populations [6, 8]. Most pathogenic *FUS* mutations cluster within the nuclear localization sequence (NLS), causing cytoplasmic mislocalization of the FUS protein, resulting in nuclear loss of function and cytoplasmic toxic gain of function [9, 10, 11].

FUS‐ALS is clinically heterogeneous, with age of onset serving as a key determinant of disease phenotype [8, 10, 12]. Juvenile‐onset ALS (jALS), defined as disease onset before age 25, is characterized by rapid progression and frequent bulbar involvement [12, 13, 14, 15]. *FUS* mutations are overrepresented in pediatric ALS, with specific variants such as P525L and truncating frameshift mutations being associated with particularly aggressive phenotypes, including early‐onset and exceptionally rapid progression [16, 17]. Emerging evidence suggests that jALS cases may present with premorbid learning difficulties and intellectual impairments, raising questions about potential neurodevelopmental abnormalities rather than purely neurodegenerative processes [10, 17, 18]. In contrast, adult‐onset ALS (aALS), with symptom onset at or after age 25, typically shows more variable progression rates and predominantly motor‐specific deficits [8, 11].

While a recent PET study using 2‐[^18^F]fluoro‐D‐glucose (FDG) reported preserved motor cortex metabolism in FUS‐ALS [19], multimodal neuroimaging studies combining FDG‐PET, MRI, and comprehensive neuropsychological assessment in FUS‐ALS are lacking.

Thus, the relationship between specific *FUS* variants, age of onset, cognitive phenotype, and neuroimaging signatures remains incompletely understood. Given the emergence of targeted therapies such as antisense oligonucleotide treatments (Clinical‐Trials.gov: NCT0476897 [20]), characterizing the full clinical and neuroimaging spectrum of FUS‐ALS and identifying genotype–phenotype correlations is of high relevance. Understanding the mechanisms underlying this clinical variability is critical for patient counseling, prognostication, and the development of targeted therapeutic strategies. This study investigates cognitive function, clinical progression, and structural and metabolic brain changes in FUS‐ALS patients stratified by age of onset, to determine whether juvenile‐ and adult‐onset forms represent distinct clinical and biological entities.

## 2 Participants and Methods

### 2.1 Participants

This retrospective study was conducted at the University Medical Center Rostock and includes patients with genetically confirmed *FUS* mutation. Patients were recruited between 2018 and 2025 and classified as juvenile‐onset ALS (jALS; disease onset < 25 years) or adult‐onset ALS (aALS; disease onset ≥ 25 years). In total, 8 participants with *FUS* mutations were included in this study, comprising 3 jALS cases and 5 aALS cases. All patients fulfilled diagnostic criteria according to the revised El Escorial (Awaji) criteria [21, 22], including two early symptomatic cases (aALS_1, aALS_3) with electromyographically supported diagnosis.

Clinical assessment included neurological examination, disease characterization using the revised ALS Functional Rating Scale (ALSFRS‐R) [23], and collection of blood plasma and cerebrospinal fluid (CSF) with analysis of neurodegeneration markers. Demographics and disease‐related parameters (Table 1) were systematically recorded, including age at onset (AaO), disease duration, site of onset (bulbar/spinal), body mass index (BMI), and occurrence of severe clinical events. ALSFRS‐R was assessed at serial visits, and progression rate (ΔALSFRS‐R) was calculated as the decline per month from symptom onset. Severe events were defined as initiation of percutaneous endoscopic gastrostomy (PEG) feeding, invasive ventilation, or death. Respiratory failure was documented based on clinical assessment and vital capacity measurements. Family history (familial vs. sporadic ALS) was obtained from all participants. Selected patients were enrolled in the FUSION trial (ION363‐CS1), a phase 3 study investigating antisense oligonucleotide treatment for FUS‐ALS (marked in Table 1).

**TABLE 1:** Clinical patient characteristics and demographics of all FUS‐ALS patients.

	Patient ID	Sex	fALS/sALS	FUS mutation	AaO (years)	Disease duration (month)^d^	Site of onset	ΔALS‐ FRS‐R	Severe event (month)^b^	Respiratory failure^c^	BMI^e^ (kg/m^2^)
jALS	jALS_1	M	sALS	P525L; c.1574C > T	23	> 15	Right leg	−1.56	10	Yes	22
	jALS_2	F	sALS	P525L; c.1574C > T	16	26^†^	Bulbar	−1.08	11	Yes	23
	jALS_3^a^	F	sALS	H517_Q519del; c.1549_1557del	23	18^†^	Left arm	−2.00	16	Yes	13
Adult‐onset ALS	aALS_1^a^	F	fALS	R514T; c.1541 G > C	39	15	Right arm	0.00	No	No	21
	aALS_2^a^	F	fALS	R514T; c.1541 G > C	39	32	Right arm	−0.52	No	Yes	41
	aALS_3^a^	F	fALS	R521C; c.1561C > T	41	84	head	0.00	No	No	22
	aALS_4	M	sALS	VUS; c.33‐66C > T	67	32^†^	Right leg	−0.55	29^f^	No	19
	aALS_5^a^	M	sALS	K510R; c.1529A > G	72	109	Left arm	−0.28	No	No	21
Cliff's Delta	—	0.07	−0.6***	—	−1.00***	−0.67***	−0.33*	−1.00***	−1.00***	0.80***	0.00

Abbreviations: aALS, adult‐onset ALS; AaO, age at onset; BMI, body mass index; fALS, familial ALS; jALS, juvenile ALS; sALS, sporadic ALS; VUS, variant of unknown significance.

^a^ Patients in the FUSION study (NCT0476897).

^b^ Severe event: PEG placement, tracheostomy or death.

^c^ Respiratory failure defined as vital capacity < 50% of predicted and/or diagnosed respiratory insufficiency (partial or global).

^d^ Data collection: 2018–2025.

^e^ BMI was recorded at the last available clinical visit.

^f^ Assisted suicide.

^†^ Dead.

* 0.14–0.33 = small effect.

*** > 0.47 = large effect.

For statistical analyses, because of the small sample sizes (jALS *n* = 3; aALS *n* = 5), Cliff's delta, a non‐parametric effect size measuring the degree of overlap between two distributions, was calculated to quantify group differences in patient characteristics using the effsize package (Version 0.8.1) in R (version 4.3.0, R Core Team, 2024). Cliff's delta ranges from −1 to +1, where positive values indicate higher values in the jALS group and negative values indicate lower values in jALS.

### 2.2 Genetic Analysis

Genetic testing was performed as part of clinical diagnosis from EDTA blood samples at different certified laboratories. Seven participants underwent targeted next‐generation sequencing using comprehensive ALS‐specific gene panels. Sequence variants were classified according to ACMG/ACGS guidelines. For aALS_3, who carried a known familial *FUS* mutation, targeted Sanger sequencing of the specific familial variant c.1561C>T (p.Arg521Cys) was performed.

**FIGURE 1:**
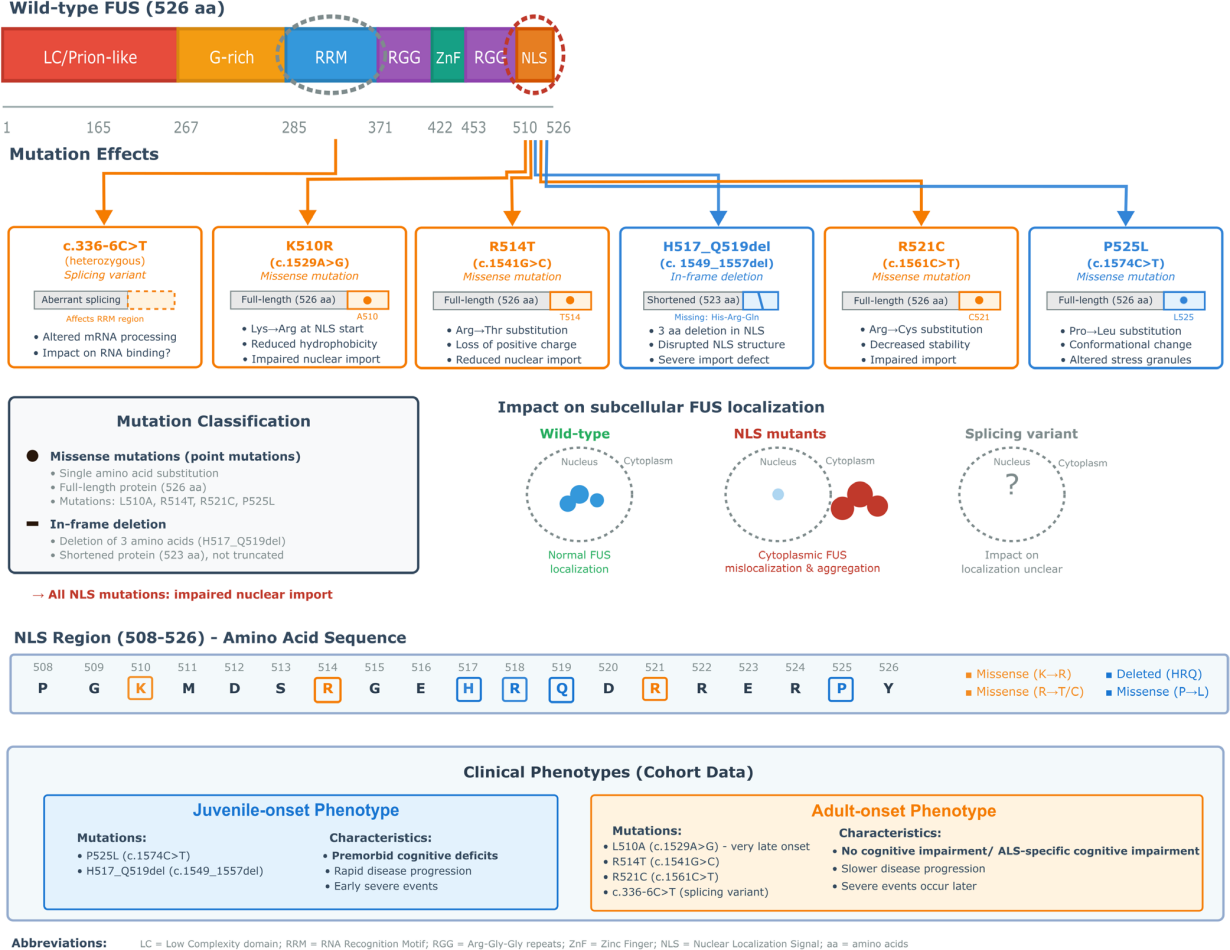
FUS protein structure and mutation landscape in juvenile‐ versus adult‐onset FUS‐ALS. Schematic representation of wild‐type FUS protein (526 amino acids) showing functional domains and patient‐specific mutations. The C‐terminal nuclear localization signal (NLS) region is critical for nuclear import. Patient mutations include one splicing variant (c.336‐6C> T), one in‐frame deletion (H517_Q519del), and four NLS missense mutations (K510R, R514T, R521C, P525L). Genotype–phenotype correlation reveals two distinct clinical subtypes: Juvenile‐onset ALS (blue) with severe NLS‐disrupting mutations (P525L, H517_Q519del) shows premorbid cognitive deficits, rapid progression, and severe events within 1 year. Adult‐onset ALS (orange) with milder NLS mutations (K510R, R521C, R514T) and splicing variant (c.336‐6C>T) demonstrates no premorbid cognitive impairment and slower disease progression without severe events over 2 years. The middle panel illustrates mutation‐dependent subcellular FUS mislocalization. Abbreviations: Aa, amino acids; LC, low complexity; RRM, RNA recognition motif; RGG, arginine‐glycine–glycine repeats; ZnF, zinc finger; NLS, nuclear localization signal.

Seven patients carried pathogenic or likely pathogenic variants, while one patient (aALS_4) had a variant of uncertain significance (VUS) (Table 1). Schematic visualization of *FUS* gene structure and mutation locations (Figure 1) was created using Inkscape (version 1.4).

### 2.3 Neuropsychological Assessment

Neuropsychological data were available for 7 of 8 participants. Premorbid verbal‐crystallized intelligence was estimated using the German Mehrfachwahl‐Wortschatz‐Test (MWT) [24], which correlates highly with general intelligence [25]. For participant jALS_1, premorbid intelligence was additionally assessed using subtests from Wechsler adult intelligence scale (“Similarities”, “Arithmetic”, “Block Design”) [26].

Current cognitive performance was assessed using the German version of the “Edinburgh Cognitive and Behavioral ALS Screen” (ECAS) [27, 28]. The ECAS comprises 15 subtests across 5 cognitive domains: ALS specific functions (verbal fluency, executive functions including social cognition, and language), and ALS‐non‐specific functions (memory and visuospatial abilities). We analyzed scores for each subdomain, the ALS‐specific total, the ALS‐nonspecific total, and the ECAS total score. Social cognition was assessed with Mini Social Cognition and Emotional Assessment (Mini‐SEA) [29], which includes the Faux Pas test and facial emotion recognition test (FRT). Specific linguistic abilities were measured by a confrontation naming task (Magdeburg Picture Naming Test, MPNT, unpublished) as well as semantic knowledge task (Kaffee & Kuchentest, K&K) [30]. Patients were cognitively classified according to the revised Strong criteria [31] as either cognitively non‐impaired (ALS‐ni) or cognitively impaired (ALS‐ci). Cognitive impairment was defined as a z‐scores ≤ −2, calculated using age‐ and education matched German normative data [28]. Data were visualized using ggplot2 in R and GraphPad Prism (version 8.2.1).

### 2.4 Structural MRI and Volumetric Analysis

Structural MRI was performed in all 8 participants using the 3.0 T Siemens Skyra Fit scanner (2018–2022) or the 3.0 T Siemens Magnetom Vida scanner (from 2022 onwards) at the Rostock University Medical Center (Rostock, Germany). High‐resolution 3D T1‐weighted images were acquired using the magnetization‐prepared rapid gradient echo (MPRAGE) sequence with the following parameters: repetition time = 2500 ms, field of view = 256 × 256 mm^2^, 192 sagittal slices, slice thickness = 1.0 mm, resulting in an isotropic voxel size of 1 mm^3^. Echo time (TE) varied slightly between subjects (2.58–4.37 ms) owing to protocol adjustments and scanner‐related differences across acquisitions. Volumetric analysis was conducted using mdbrain software (mediaire GmbH, Berlin, Germany), which provides deep learning‐based segmentation and quantification of brain structures including cortical and subcortical regions. Volumetric values were expressed as age‐ and sex‐adjusted percentiles relative to a normative reference database to identify atrophy patterns [32].

### 2.5 FDG‐PET Imaging and Analysis

Brain FDG‐PET imaging was performed in 4 of 8 participants using a Gemini TF 16 PET/CT scanner (Philips Healthcare, Best, Netherlands). Following intravenous injection of 199 ± 18 MBq FDG, patients rested for 30 min before undergoing dynamic PET acquisition consisting of four consecutive 5 min frames. An auxiliary CT scan (120 kVp, 30 mAs) was acquired prior to PET imaging for attenuation correction. To ensure image quality, each dynamic frame was visually inspected for patient motion artifacts. Frames showing significant movement were excluded from further analysis. The remaining frames (representing at least 10 min of acquisition time) were combined into a static PET dataset and reconstructed using the vendor‐specific BLOB‐OS algorithm (3 iterations, 31 subsets) with corrections applied for randoms, scatter, decay, and attenuation based on CT‐derived information.

Analysis and visualization of regional brain hypometabolism were performed using NEUROSTAT software (University of Washington, Seattle, USA) which applies three‐dimensional stereotactic surface projection (3D‐SSP) to compare cortical activity with an age‐matched normative database while reducing the influence of cortical atrophy. Additionally, quantitative regional assessment of glucose metabolism with age‐matched z‐scores was conducted using BRASS (Brain Registration and Analysis Software Suite; Hermes Medical Solutions AB, Stockholm, Sweden), which uses atlas‐based spatial normalization and predefined volumes of interest to generate regional uptake values and corresponding age‐matched z‐scores.

## 3 Results

### 3.1 Clinical Characteristics, Demographics and Biomarker Profiles

Detailed clinical characteristics and disease progression metrics for all patients are presented in Table 1 and Figure S1, while fluid and electrophysiological biomarker profiles are summarized in Table 2.

**TABLE 2:** Fluid biomarker profile and electrophysiology of patients.

	Patient ID	Fluid biomarker				Electrophysiology	
		Serum NfL (pg/mL)^a^ ^,^ ^b^ [*z‐score or ref*]	CSF NfL (pg/mL)^a^ [*z‐score or ref*]	CSF pNfH (pg/mL)^a^, ^c^	AD marker	ENG	EMG
jALS	jALS_1	318 [*ref:* < 25]	N/A	N/A	Borderline^d^	axonal neuropathy + F‐wave abnormalities	widespread denervation (> 3 regions), chronic changes
	jALS_2	N/A	N/A	4257	N/A	normal	widespread denervation (> 3 regions)
	jALS_3	N/A	N/A	N/A	normal	axonal damage ulnar/median left	widespread denervation (> 3 regions)
Adult‐onset ALS	aALS_1	28 [1.84]	N/A	N/A	normal	N/A	minimal fasciculations, otherwise normal
	aALS_2	74 [> 2.5]	1464*[2.32]*	N/A	normal	normal	denervation 2 muscles (deltoid, trapezius left), early recruitment
	aALS_3	74 [ref: < 25]	N/A	987	N/A	normal	fasciculations (IOD1 left, tibialis ant right), no denervation
	aALS_4	195 [> 2.5]	8072.2[*ref:* 762]	N/A	normal	F‐wave loss, distal normal	N/A
	aALS_5	47 [0.44]	4431[1.88]	N/A	tau/ptau↑; A*β* normal	N/A	N/A

Abbreviations: aALS, adult‐onset ALS; AD, Alzheimer Disease; Aβ, Amyloid β CSF, cerebrospinal fluid; EMG, Electromyography; ENG, Electroneurography; jALS, juvenile ALS; N/A, not assessed; NfL, Neurofilament light chain; pNfH, phosphorylated neurofilament heavy chain.

^a^ NfL/pNfH was measured at the closest available timepoint to initial clinical presentation and prior to study medication, except for aALS_5 (FUSION trial, later timepoint under established study medication).

^b^ Reference < 40 years: < 25 pg/mL, > 40 years: < 45 pg/mL.

^c^ Reference: < 560 pg/mL.

^d^ The pathological value (*β*42/*β*40 Ratio) is considered to be artefactual.

Cliff's Delta analysis revealed negligible effect sizes between jALS and aALS for sex distribution and body mass index (BMI; δ = 0.000), while a medium effect size was observed for site of disease onset (δ = −0.333), reflecting one bulbar‐onset patient in the jALS group compared to spinal onset in all aALS patients. However, marked group differences with large effect size in disease severity and progression were observed. jALS patients exhibited more aggressive disease trajectories compared to aALS patients, with ΔALSFRS‐R values ranging from −1.08 to −2.00 (Figure S1). Furthermore, jALS patients experienced early‐onset respiratory failure and severe disease complications, ultimately resulting in premature mortality. In contrast, the aALS cohort demonstrated a more benign clinical course. Except for one patient who elected physician‐assisted suicide, no severe adverse events were documented in the aALS group. Only one aALS patient (aALS_2) developed respiratory insufficiency during the observation.

Alzheimer disease biomarkers exhibited no phenotype‐specific pattern, with normal to borderline values observed in both groups (Table 2, Table S1). Neurofilament measurements (serum and CSF NfL, pNfH) were available in a subset of patients (Table 2). Among evaluable cases, no phenotype‐specific differences between jALS and aALS were evident.

Electrophysiological assessments (ENG, EMG) were performed in a subset of patients, with considerable variability in data completeness (Table 2). EMG revealed more widespread denervation in jALS compared to aALS patients, while ENG findings ranged from normal to axonal neuropathy and F‐wave abnormalities. However, incomplete data precluded systematic between‐group comparisons. Overall, no consistent phenotype‐specific electrophysiological signatures could be identified to distinguish jALS from aALS in this cohort.

### 3.2 FUS Mutation Spectrum and Genotype‐Associated Phenotypes

Genetic testing revealed pathogenic *FUS* mutations in seven patients, while patient aALS_4 had a variant of uncertain significance (VUS) in the *FUS* gene (Figure 1, Table 1).

The three jALS patients harbored de novo mutations within the C‐terminal NLS domain (amino acids 495–526): two patients carried the recurrent P525L mutation located in the proline‐tyrosine (PY) motif essential for nuclear import, while one patient (jALS_3) presented with the novel in‐frame deletion H517_Q519del. All jALS cases were sporadic.

Among the five aALS patients, four carried pathogenic mutations also located within the NLS region (R514T, R521C, K510R). The fifth aALS patient (aALS_4) carried a splicing variant (VUS) located outside the NLS domain, highlighting the phenotypic heterogeneity associated with non‐canonical *FUS* mutations. Familial inheritance was observed in 60% of aALS cases (3/5) but in none of the jALS cases (0/3), showing a large effect size in Cliff's Delta analysis (δ = −0.6, Table 1).

### 3.3 Neurocognitive Profiles

Cognitive assessment included premorbid records, neuropsychological assessments, and classification according to Strong criteria [31].

**FIGURE 2:**
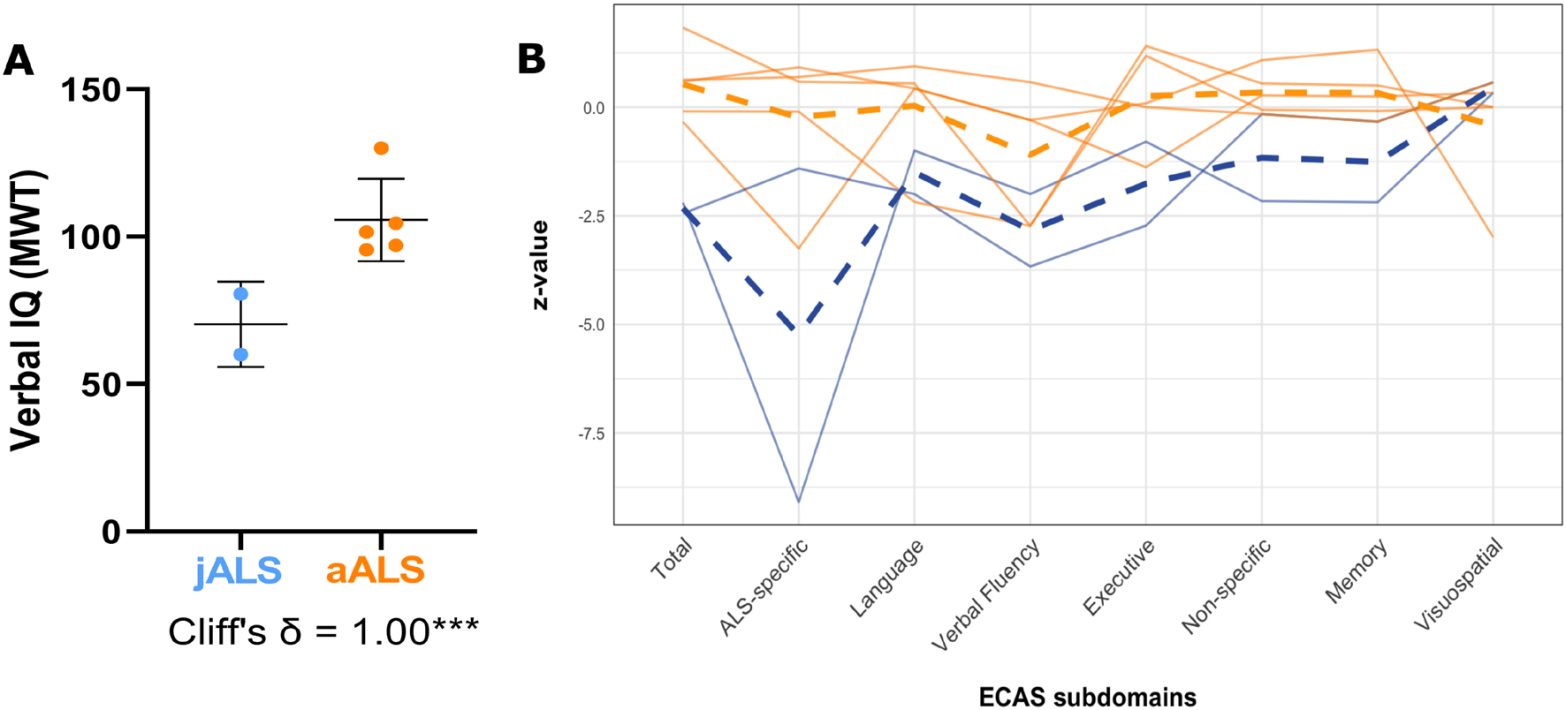
Neuropsychological assessment reveals distinct cognitive profiles in juvenile‐ versus adult‐onset FUS‐ALS. (A) Verbal IQ assessed by MWT (Mehrfachwahl‐Wortschatz‐Test) demonstrates significantly reduced crystallized intelligence in jALS (blue, *n* = 2) compared to aALS (orange, *n* = 5). Individual data points with mean ± SD. Cliff's δ = 1.00, indicating large effect size (***p < 0.001). (B) ECAS (Edinburgh Cognitive and Behavioral ALS Screen) z‐scores across cognitive domains for FUS‐ALS patients grouped by disease onset. Juvenile‐onset patients (blue lines, *n* = 2) demonstrate global cognitive deficits across all ECAS domains, consistent with premorbid intellectual disability or learning difficulties. Adult‐onset patients (orange lines, *n* = 4) show predominantly preserved cognition with mild ALS‐specific impairments in selected cases. Bold dashed lines represent group averages for jALS (blue) and aALS (orange). Abbreviations: ECAS, Edinburgh Cognitive and Behavioral ALS Screen; MWT, Mehrfachwahl‐Wortschatz‐Test; jALS, juvenile‐onset ALS; aALS, adult‐onset ALS; ALS‐ci, ALS with cognitive impairment.

Premorbid cognitive impairments were documented in all three jALS patients, supported by reduced verbal IQ on the MWT in the two patients assessed, pointing towards premorbid cognitive impairment predating ALS motor onset. Among the two patients carrying the P525L mutation, one had been diagnosed with intellectual disability (ICD‐10: F71) prior to ALS motor onset, while the other had a documented specific developmental disorder of scholastic skills (ICD‐10: F81.2). The third jALS patient (H517_Q519del) attended regular schooling but exhibited mild learning difficulties, with premorbid intelligence assessed by MWT placing her in the lower average range (IQ 80.5). In contrast, all aALS patients scored markedly higher, with a Cliff's δ of 1.00 indicating maximum effect size separation between groups (Figure 2A).

Cognitive assessment data were available for two of the three jALS patients. Both demonstrated impairments across all domains of the ECAS (blue lines in Figure 2B), meeting criteria for ALS‐ci. Notably, the pattern reflected global cognitive deficits rather than domain‐specific ALS‐associated impairments. Additional deficits were observed in confrontation naming, MPNT, while semantic object knowledge remained preserved (Table 3). In the social cognition domain, only one jALS patient completed the Faux Pas Recognition Test and obtained a low score. However, this test requires relatively high abilities in verbal comprehension. Emotion recognition from facial expressions was unremarkable in all ALS patients (Table 3). In the aALS cohort, two patients exhibited isolated reductions in verbal fluency (orange lines in Figure 2; Table 3), an ALS‐specific cognitive marker, and were classified as ALS‐ci. The remaining aALS patients were cognitively unimpaired (ALS‐ni).

**TABLE 3:** Cognitive scores of patients.

	Patient ID	Verbal IQ	ECAS								Language		Social cognition	
		MWT‐B	Total score	ALS‐specific	Language	Verbal fluency	Executive	ALS‐nonspecific	Memory	Visuo‐spatial	MPNT	K&K	Faux‐pas test	FRT
jALS	jALS_1	60^a^	−2.5	−1.4	−2.0	−3.7	−2.7	−0.2	0.6	−0.3	−3.4	−0.1	n.a.	10.7
	jALS_2	n.a.	n.a.	n.a.	n.a.	n.a.	n.a.	n.a.	n.a.	n.a.	n.a.	n.a.	n.a.	n.a.
	jALS_3	80.5	−2.2	−9.1	−1.0	−2.0	−0.8	−2.2	0.3	−2.2	−2	1.5	7.6	12.9
Adult‐onset ALS	aALS_1	104.5	0.63	0.69	0.94	0.58	0.00	−0.16	0.57	−0.33	0.9	−0.1	10.5	13.3
	aALS_2	95.5	0.6	0.9	0.4	−0.3	0.1	1.1	−3.0	1.3	0.9	0.7	15	10.7
	aALS_3	97	−0.3	−3.3	0.4	−0.30	−1.4	0.3	0.3	0.2	0.9	0.7	12	10.7
	aALS_4	130	1.8	0.6	0.6	−2.7	1.4	0.6	0.0	0.5	0.9	−0.1	10.9	10.7
	aALS_5	101.5	−0.1	−0.1	−2.2	−2.7	1.2	−0.1	0.0	−0.1	−0.6	−0.1	10.1	13.3
Cliff's Delta		1.00***	1.00***	−0.39***	−0.18**	−0.80***	−0.12*	−0.31**	−0.31***	0.02*	0.18**	−0.31**	1.00***	1.00***

Abbreviations: aALS, adult‐onset ALS; ECAS, Edinburgh Cognitive and Behavioral ALS Screen; FRT, Face Recognition Test; jALS, juvenile ALS; K&K, Kaffee & Kuchentest; MPNT, Magdeburg Picture Naming Test; MWT‐B, Mehrfachwahl‐Wortschatztest.

^a^ Verbal IQ was assessed by Wechsler Intelligence Scale‐III: Subtest Similarities.

* 0.14–0.33 = small effect.

** 0.33–0.47 = medium effect.

*** > 0.47 = large effect.

Group comparison using Cliff's delta revealed at least moderate effect sizes for nearly all cognitive variables (Table 3), with jALS patients performing worse than aALS patients in almost all areas. In particular, ALS‐nonspecific and visuospatial scores in the ECAS were affected to a similar extent compared to ALS‐specific scores. Overall, jALS patients exhibited generalized cognitive deficits consistent with premorbid intellectual disability or learning difficulties.

### 3.4 Multimodal Neuroimaging Findings

**FIGURE 3:**
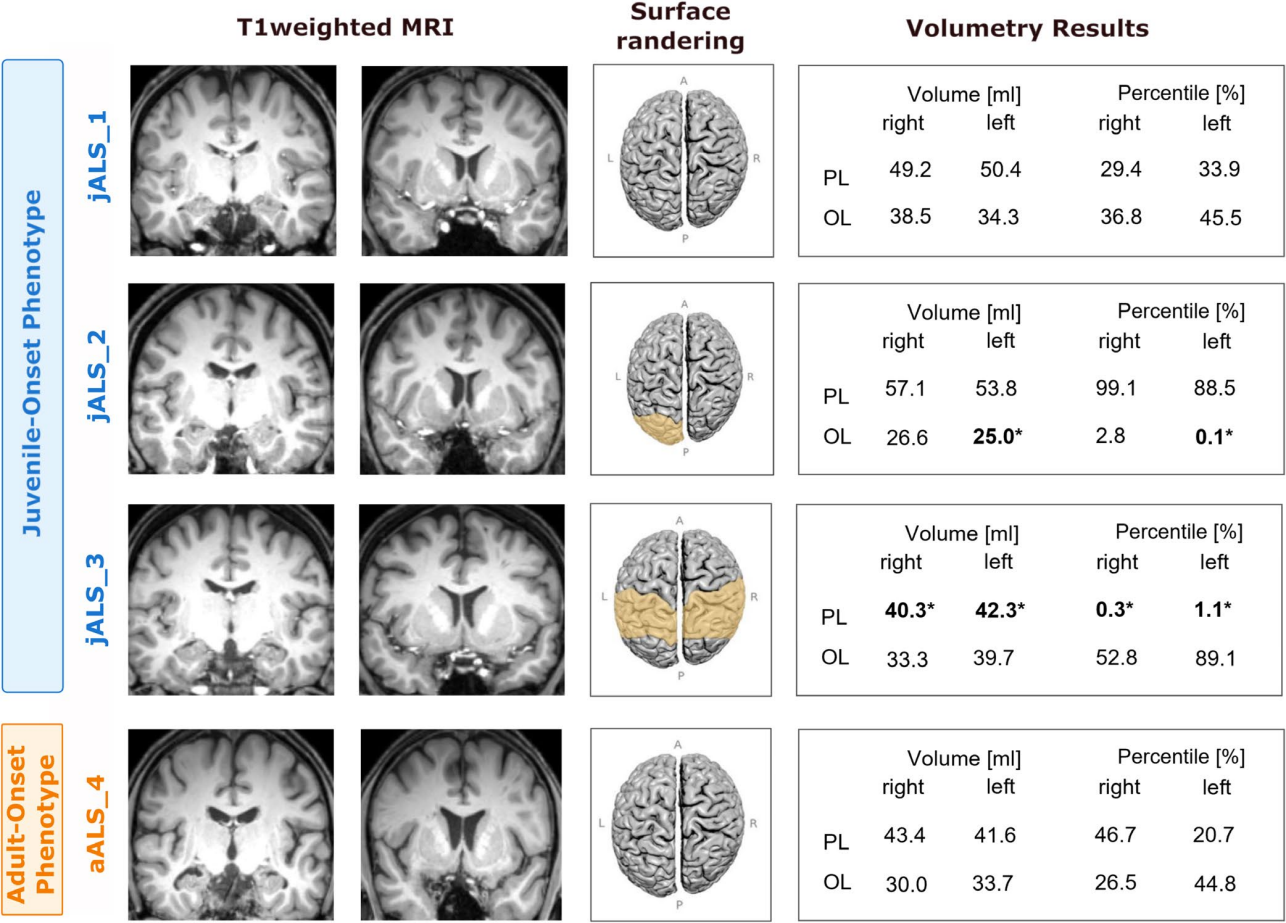
Patterns of cortical atrophy in jALS vs. aALS. Representative T1‐weighted MRI with volumetric analysis and 3D surface rendering for all three jALS patients (blue) and one aALS patient (aALS_4; orange). Volumetric results display absolute volumes (mL) and age‐and sex‐adjusted percentiles for parietal lobe (PL) and occipital lobe (OL). * indicates pathologically reduced volumes (< 5th percentile). jALS patients demonstrate posterior cortical atrophy with predominant involvement of occipital and parietal regions (orange areas in surface rendering): JALS_2 shows severe bilateral occipital atrophy (left OL: 0.1st percentile), while jALS_3 exhibits bilateral parietal atrophy (bilateral PL: 0.3–1.1st percentiles). In contrast, aALS patients show preserved cortical volumes across all regions, with aALS_4 shown here as a representative example of the aALS phenotype with no pathological atrophy detected. Abbreviations: PL, parietal lobe; OL, occipital lobe; jALS, juvenile‐onset ALS; aALS, adult‐onset ALS; L, left; R, right; A, anterior; P, posterior.

T2‐weighted MRI sequences revealed small, nonspecific white matter hyperintensities in some patients from both groups. Volumetric analysis using the mdbrain algorithm demonstrated distinct patterns of brain atrophy between phenotypes (Figure 3, Figure S2). Among jALS patients, two of three cases (67%) exhibited significant age‐matched cortical atrophy with posterior predominance: jALS_2 showed marked occipital lobe atrophy (left hemisphere volume at 0.1st percentile, *p* < 0.05), while jALS_3 demonstrated profound bilateral parietal lobe atrophy (right 0.3rd percentile, left 1.1st percentile, *p* < 0.05). In contrast, jALS_1 showed preserved brain volumes across all regions. The aALS cohort demonstrated largely preserved cortical volumes (for one representative patient see Figure 3), with only one patient (aALS_1, 20%) exhibiting focal left parietal lobe atrophy (2.0th percentile, *p* < 0.05; Figure S2). No patient in either group showed significant cerebellar or brainstem atrophy. Overall, neuroimaging revealed distinct patterns between phenotypes: jALS cases demonstrated posterior cortical atrophy in the majority of cases, while aALS patients showed largely preserved brain volumes.

**FIGURE 4:**
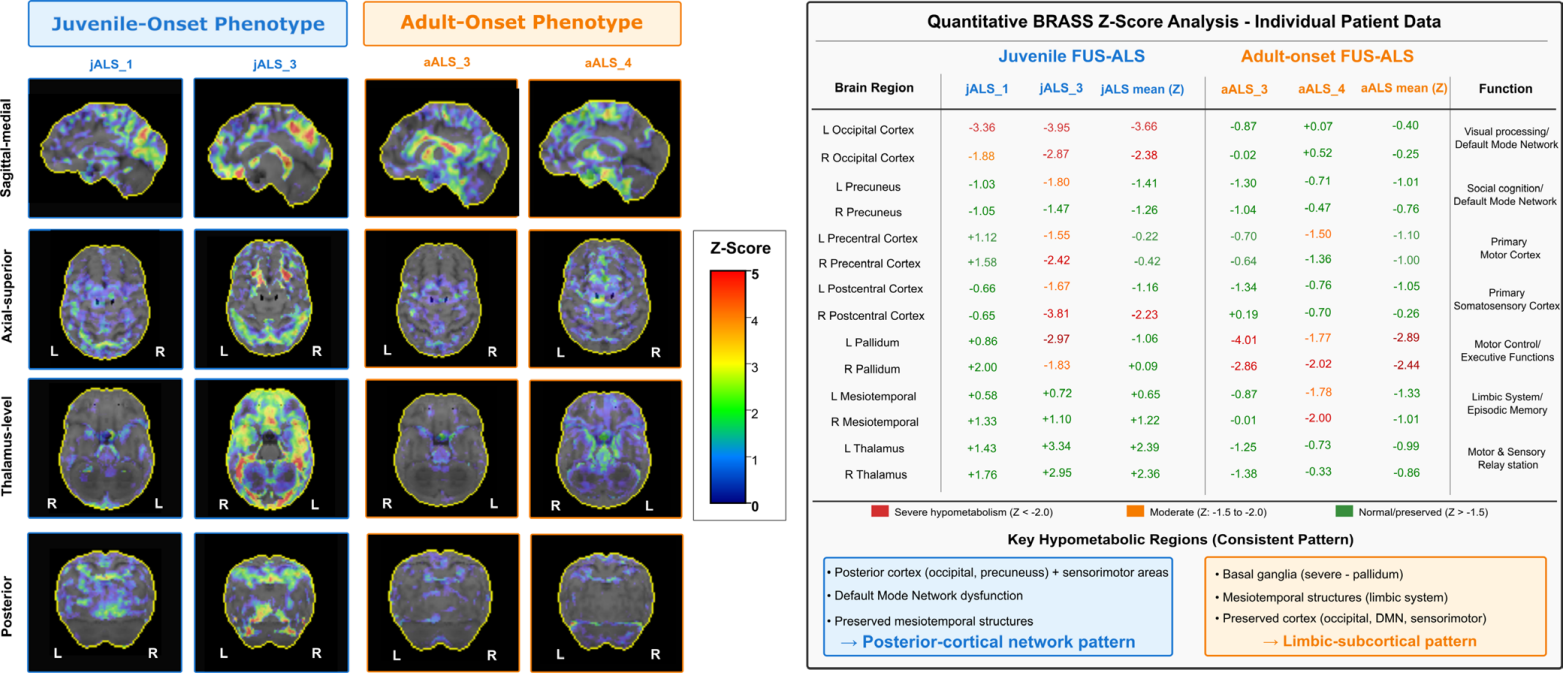
FDG‐PET reveals distinct metabolic patterns in jALS vs. aALS FUS‐ALS. (Left panel) Representative FDG‐PET z‐score maps for two juvenile‐onset patients (jALS_1, jALS_3; blue frame) and two adult‐onset patients (aALS_3, aALS_4; orange frame) displayed in sagittal‐medial, axial‐superior, thalamus‐level, and posterior views. Color scale represents regional metabolism z‐scores relative to age‐matched controls; warm colors (red‐orange) indicate hypometabolism, cool colors (blue) indicate normal or hypermetabolism. (Right panel) Quantitative BRASS (Brain Abnormality by Statistical Segmentation) z‐score analysis for individual patients across functionally relevant brain regions. Z‐scores < −2.0 (red) indicate severe hypometabolism, −2.0 to −1.5 (orange) moderate hypometabolism, and > −1.5 (green) normal/preserved metabolism. jALS patients demonstrate severe posterior‐cortical hypometabolism while aALS patients show predominantly limbic‐subcortical hypometabolism with relative preservation of cortical metabolism. Abbreviations: FDG‐PET, fluorodeoxyglucose positron emission tomography; BRASS, Brain Abnormality by Statistical Segmentation; L, left; R, right; jALS, juvenile‐onset ALS; aALS, adult‐onset ALS.

Recent FDG‐PET studies have characterized metabolic patterns in FUS‐ALS, demonstrating relative preservation of motor cortex metabolism compared to sporadic ALS [19]. To further investigate metabolic signatures within FUS‐ALS phenotypes and correlate them to clinical assessments, FDG‐PET imaging was performed for a subset of patients (2 jALS, 2 aALS, Figure 4). Neurostat and BRASS analysis revealed that both jALS patients demonstrated posterior‐cortical hypometabolism, most pronounced in patient jALS_3, who exhibited severe bilateral occipital (z‐score: L‐3.95; *R* −2.87) and postcentral cortex (z‐score: L‐1.67; *R* −3.81) hypometabolism, extending into precentral regions (z‐score: L‐1.55; *R* −2.42). The occipital glucose hypometabolism distinguished jALS from aALS, in whom occipital metabolism was relatively preserved (mean z‐score: L‐0.40; *R* −0.25 vs. L‐3.66; R −2.38 in jALS).

In contrast, the aALS cohort exhibited a limbic‐subcortical hypometabolic pattern. Both patients demonstrated basal ganglia hypometabolism (mean z‐score: L‐2.89; *R* −2.44), while mesiotemporal involvement was predominantly seen in aALS_4 (z‐score: L‐1.78; *R* −2.00). Cortical metabolism remained largely intact.

Distinct metabolic patterns emerged between phenotypes: jALS demonstrated predominantly posterior‐cortical metabolic deficits, while aALS showed subcortical‐limbic involvement (Figure 4).

## 4 Discussion

FUS‐associated ALS represents a genetically defined subgroup of motor neuron disease characterized by considerable phenotypic heterogeneity, ranging from aggressive juvenile‐onset forms to more slowly progressive adult‐onset disease [8, 11, 12]. In this study, we provide comprehensive clinical, genetic, neuropsychological, and multimodal neuroimaging characterization of eight FUS‐ALS patients, demonstrating distinct phenotypic profiles between jALS and aALS cases.

The aggressive clinical course in our jALS patients, characterized by rapid progression and early severe events, aligns with the established correlation in FUS‐ALS that younger age at disease onset predicts both greater disease severity and accelerated time to critical complications [12]. More recently, Grassano et al., (2022) refined this genotype–phenotype relationship by identifying three distinct FUS‐ALS phenotypic clusters arising from different mutations: (1) axial ALS with upper cervical and dropped‐head onset in mid‐to‐late adulthood; (2) benign ALS, with late onset and slow disease progression; and (3) juvenile ALS, frequently preceded by learning disability or mild intellectual disability (47.4%) or presenting with bulbar involvement (36%). In our cohort, all aALS patients aligned with Cluster 2 (benign ALS), while all jALS patients matched Cluster 3 (juvenile ALS). No axial phenotype was observed.

Consistent with this previously described jALS phenotype, neuropsychological assessment in our cohort revealed moderate to large effect sizes (Cliff's Delta) across multiple cognitive domains between jALS and aALS patients.

The pattern of premorbid cognitive impairment represents a characteristic feature of severe FUS‐ALS but is not consistently reported in classical ALS cohorts. Multiple case reports support this association: patients with severe truncating *FUS* mutations (G504Wfs*12, Q519IfsX527, G505Wfs*12) and P525L mutations consistently presented with both juvenile‐onset ALS and intellectual disability or mental retardation [16, 17, 33]. More recently, Verma et al. (2023) [34] employed a multiomic approach in a 14‐year‐old patient carrying the D490Gfs*26 frameshift *FUS* mutation with learning disabilities, finding no pathogenic variants in established intellectual disability associated genes and thereby supporting FUS dysfunction as the primary driver of the cognitive phenotype. However, in our cohort, genetic testing was performed using a targeted ALS‐specific gene panel rather than whole genome sequencing, precluding systematic analysis of intellectual disability‐associated genes or potential oligogenic contributions as proposed by Goldstein et al. (2022). Thus, whether additional genetic modifiers contribute to the cognitive phenotype in our jALS patients remains to be determined.

A systematic literature review of 175 FUS‐ALS cases from Goldstein et al. (2022) [18] showed that P525L carriers had significantly higher rates of intellectual disability compared to other *FUS* missense mutation carriers (19% vs. 1.6%, *p* = 0.0135), with affected individuals showing earlier age at onset and shorter disease duration. Consistent with this, both of our jALS patients with P525L mutation presented with premorbid intellectual disability or learning difficulties. The P525L mutation disrupts the PY motif critical for Karyopherin *β*2 binding, severely impairing nuclear import [35]. The third patient in our jALS cohort carried the novel H517_Q519del mutation, represents an in‐frame deletion of three consecutive amino acids (positions 517–519) within the NLS domain. This deletion, although located upstream of the PY motif, is predicted to cause severe structural disruption of the entire NLS region. Unlike single amino acid substitutions at similar positions (e.g., R521C, R514T), which have been associated with milder, adult‐onset phenotypes [8, 12], multi‐residue deletions fundamentally alter the three‐dimensional architecture of the NLS, potentially disrupting the spatial arrangement required for Karyopherin *β*2 recognition and binding. This structural perturbation likely interferes with the proper presentation of the downstream PY motif, resulting in severe impairment of nuclear import comparable to that observed with P525L mutations. Both mutations are therefore predicted to cause substantial cytoplasmic FUS mislocalization, consistent with the severe juvenile‐onset phenotype observed in these patients. In contrast to jALS, the aALS patients predominantly carried NLS mutations (R514T, R521C, K510R) predicted to cause less severe nuclear import impairment compared to jALS‐associated mutations, consistent with their milder, adult‐onset phenotype [8, 12]. This observation aligns with the fundamental genotype–phenotype correlation in FUS‐ALS: the degree to which NLS mutations impair nuclear import, thereby leading to cytoplasmic FUS mislocalization, correlates with the age of disease onset [8, 36, 37].

Notably, intellectual disability in our juvenile FUS‐ALS cases manifested years before motor symptom onset, suggesting neurodevelopmental disruption rather than early neurodegeneration. Similar observations have been made in presymptomatic *C9orf72* expansion carriers [38], who demonstrate stable verbal fluency deficits and structural white matter alterations prior to motor symptom onset. Crucially, these deficits showed no progression over 12 months and did not correlate with disease duration, arguing against active neurodegeneration and instead supporting a neurodevelopmental origin. Together, these findings suggest that certain ALS‐associated mutations, including C9orf72 and potentially FUS variants, may exert effect early in neurodevelopment. Beyond its established roles in mature neurons, accumulating evidence indicates that FUS is critically involved in neurodevelopmental processes, particularly those governing synaptic function and the structural organization of the central nervous system [10, 39]. Our multimodal neuroimaging findings are consistent with this neurodevelopmental hypothesis. Strikingly, our FUS‐ALS cohort revealed contrasting patterns of brain involvement: jALS was characterized by posterior cortical atrophy and hypometabolism affecting predominantly occipital and parietal regions, whereas aALS showed largely preserved cortical volumes accompanied by subcortical‐limbic metabolic dysfunction. While posterior involvement may seem unexpected, Agosta et al. (2012) previously demonstrated gray matter reductions extending beyond motor regions to parietal and occipital cortices in ALS [40], lending support to our findings in juvenile FUS‐ALS.

Cognitive impairment in ALS correlates with frontotemporal atrophy on neuroimaging, with structural changes often present at diagnosis [31, 41, 42]. Frontal cortical involvement has been documented particularly in FUS‐associated cases, where cognitive deficits correlate with structural brain abnormalities on MRI [14, 43]. However, existing case reports described cognitive impairment in FUS‐ALS, also in P525L, in the absence of overt structural brain atrophy on MRI [16, 33, 44].

The observed posterior‐predominant pattern in our jALS with *FUS* mutations contrasts sharply with the frontotemporal degeneration typically observed in adult‐onset ALS [31, 45] and differs from the largely preserved cortical volumes with subcortical‐limbic metabolic dysfunction observed in our adult‐onset FUS‐ALS cases. While Tan et al. (2022) [46] recently identified a cingulate‐parietal–temporal subtype in 28% of ALS patients, our juvenile cases show an even more posteriorly shifted pattern with particularly striking occipital involvement, a region typically spared in adult ALS.

In contrast to classical neurodevelopmental intellectual disability, where MRI findings predominantly reveal structural malformations such as corpus callosum dysplasia and cortical dysgenesis [47, 48], intellectual disability in severe FUS‐ALS may reflect early neurodegenerative processes during critical developmental periods, potentially manifesting as progressive atrophic changes rather than congenital malformations. The occipito‐parietal degeneration pattern may partially account for the severe cognitive impairments and intellectual disability observed in FUS‐associated jALS, as these regions are essential for visuospatial processing, attention, and cognitive integration—functions critical for normal cognitive development.

This multimodal study suggests that severe NLS‐disrupting *FUS* mutations (P525L, H517_Q519del) may define a distinct juvenile‐onset phenotype characterized by aggressive progression, premorbid intellectual disability, and posterior cortical degeneration. The posterior‐predominant atrophy and hypometabolism, coupled with premorbid cognitive impairment, suggest neurodevelopmental dysfunction rather than classical neurodegeneration, contrasting sharply with the subcortical‐limbic patterns observed in adult‐onset FUS‐ALS. These findings further support the critical role of NLS integrity and the degree of nuclear import impairment in determining disease onset and severity in FUS‐ALS.

Limitations of our study comprise (a) small sample size, particularly for juvenile cases (*n* = 3), and (b) the retrospective nature of our analyses with some missing data, which limit statistical power and generalizability. Importantly, no inferential statistical comparisons were performed between groups due to the small sample size. All reported findings are therefore based on descriptive analyses, while the non‐parametric effect size measure Cliff's delta is provided solely to contextualize the magnitude of observed differences rather than to support inferential conclusions. Nevertheless, the consistency of findings across clinical, neuroimaging, and neuropsychological domains lends internal coherence to the observed patterns, which warrant investigation in lager cohorts. Future studies are needed to determine whether the prominent occipito‐parietal metabolic alterations observed in our juvenile FUS‐ALS cases reflect developmental‐stage‐specific vulnerability or represent a broader feature of juvenile‐onset motor neuron disease.

Our findings expand the FUS‐ALS phenotypic spectrum beyond motor neuron disease and have important clinical implications. With FUS‐targeted antisense oligonucleotide therapy (ION363/Jacifusen) currently in phase 3 clinical trials, early genetic identification of FUS‐ALS patients becomes increasingly critical. Genetic screening for *FUS* mutations should be considered in patients with intellectual disability and emerging motor symptoms, as early therapeutic intervention may be most beneficial during presymptomatic or early disease stages.

## Author Contributions

Data collecting: A.V.J., M.N., A.L., H.S., J.K., J.P., A.H. and E.K.; conceptualization: A.V.J., A.L., J.P., A.H. and E.K.; writing‐original draft preparation: A.V.J., H.S., A.H. and E.K.; writing‐review and editing: A.V.J., M.N., A.L., H.S., J.K., J.P., A.H. and E.K.; visualization: A.V.J., H.S., J.K. and E.K.; All authors contributed to the article and approved the submitted version.

## Funding

A.V.J. is supported by the Clinician Scientist Program of the Center for Transdisciplinary Neuroscience Rostock (CTNR) of the University Medical Center Rostock. A.H. is supported by the Hermann und Lilly Schilling‐Stiftung für medizinische Forschung im Stifterverband.

## Ethics Statement

The study was carried out in accordance with the ethical standards laid down in the 1964 Declaration of Helsinki and its later amendments. All participants gave their informed consent prior to study inclusion. The local ethics committees at the Rostock University Medical Centre approved the study (A2014‐162; A2024‐0023).

## Conflicts of Interest

The authors declare no conflicts of interest.

## Supporting information


**Figure S1:** Disease progression trajectories in FUS‐ALS. Progression of ALS Functional Rating Scale‐Revised (ALSFRS‐R) scores over time from disease onset. Juvenile‐onset patients (jALS_1–3, red lines) demonstrate rapid functional decline with steep trajectory slopes. Adult‐onset patients (aALS_1–5, blue lines) show slower, more variable progression patterns with preserved function over extended follow‐up periods. Y‐axis: ALSFRS‐R total score (0–48 points, where 48 represents normal function and lower scores indicate greater disability). X‐axis: time from disease onset (months). Each line represents an individual patient trajectory. Abbreviations: ALSFRS‐R, Amyotrophic Lateral Sclerosis Functional Rating Scale‐Revised; jALS, juvenile‐onset ALS; aALS, adult‐onset ALS.

**Figure S2:** Brain volumetry in aALS patients demonstrates largely preserved cortical volumes. Representative T1‐weighted MRI with volumetric analysis and 3D surface rendering for four adult‐onset FUS‐ALS patients (aALS_1, aALS_2, aALS_3, aALS_5; orange frame). Volumetric results display absolute volumes (mL) and age‐adjusted percentiles for parietal lobe (PL) and occipital lobe (OL). *indicate pathologically reduced volumes (< 5th percentile). aALS patients demonstrate largely preserved cortical volumes with only mild regional reductions: aALS_1 shows isolated left parietal atrophy (2.0th percentile), while all other patients exhibit normal to low‐normal volumes without pathological atrophy (orange areas in surface rendering represent regions below 5th percentile). Abbreviations: PL, parietal lobe; OL, occipital lobe; aALS, adult‐onset ALS; L, left; R, right; A, anterior; P, posterior.

**Table S1:** Detailed Alzheimer marker in CSF of FUS‐ALS cohort.

## Data Availability

The data that support the findings of this study are available from the corresponding author upon reasonable request.
