# Supplementary figures and images for: Cognitive and Neuroimaging Divergence Between Juvenile and Adult FUS Amyotrophic Lateral Sclerosis
Source: Ann Clin Transl Neurol. 2026 Jun 15:10.1002/acn3.70447. Online ahead of print. doi: 10.1002/acn3.70447 (PMC13394925; doi:10.1002/acn3.70447)

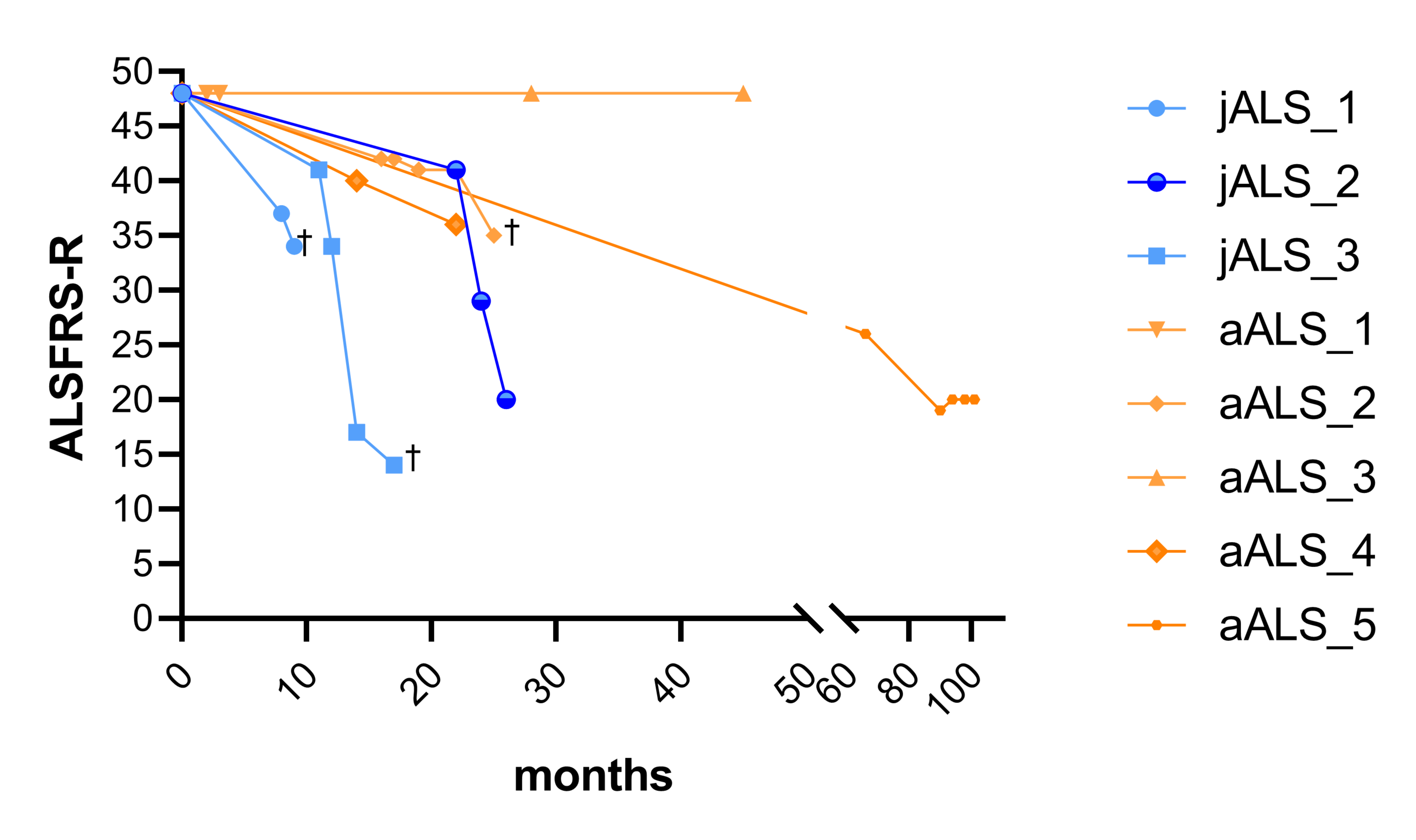

Supplement: Supplementary file 1 — Figure S1: Disease progression trajectories in FUS‐ALS. Progression of ALS Functional Rating Scale‐Revised (ALSFRS‐R) scores over time from disease onset. Juvenile‐onset patients (jALS_1–3, red lines) demonstrate rapid functional decline with steep trajectory slopes. Adult‐onset patients (aALS_1–5, blue lines) show slower, more variable progression patterns with preserved function over extended follow‐up periods. Y‐axis: ALSFRS‐R total score (0–48 points, where 48 represents normal function and lower scores indicate greater disability). X‐axis: time from disease onset (months). Each line represents an individual patient trajectory. Abbreviations: ALSFRS‐R, Amyotrophic Lateral Sclerosis Functional Rating Scale‐Revised; jALS, juvenile‐onset ALS; aALS, adult‐onset ALS. [file ACN3-9999-0-s003.png]

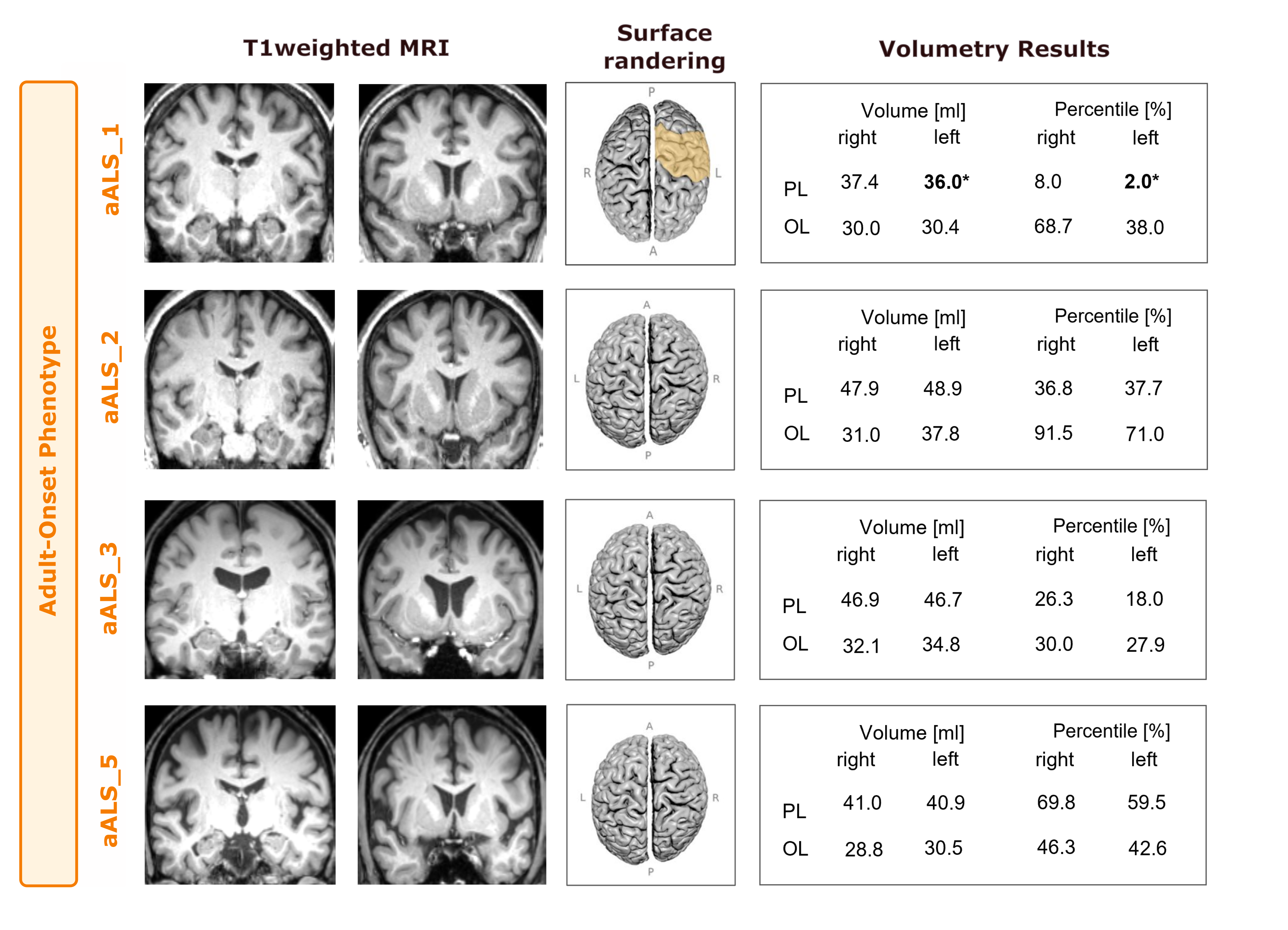

Supplement: Supplementary file 2 — Figure S2: Brain volumetry in aALS patients demonstrates largely preserved cortical volumes. Representative T1‐weighted MRI with volumetric analysis and 3D surface rendering for four adult‐onset FUS‐ALS patients (aALS_1, aALS_2, aALS_3, aALS_5; orange frame). Volumetric results display absolute volumes (mL) and age‐adjusted percentiles for parietal lobe (PL) and occipital lobe (OL). *indicate pathologically reduced volumes (< 5th percentile). aALS patients demonstrate largely preserved cortical volumes with only mild regional reductions: aALS_1 shows isolated left parietal atrophy (2.0th percentile), while all other patients exhibit normal to low‐normal volumes without pathological atrophy (orange areas in surface rendering represent regions below 5th percentile). Abbreviations: PL, parietal lobe; OL, occipital lobe; aALS, adult‐onset ALS; L, left; R, right; A, anterior; P, posterior. [file ACN3-9999-0-s002.png]
