# Supplementary material for: Cognitive and Neuroimaging Divergence Between Juvenile and Adult FUS Amyotrophic Lateral Sclerosis
Source: Ann Clin Transl Neurol. 2026 Jun 15:10.1002/acn3.70447. Online ahead of print. doi: 10.1002/acn3.70447 (PMC13394925; doi:10.1002/acn3.70447)
Supplement: Supplementary file 3 — Table S1: Detailed Alzheimer marker in CSF of FUS‐ALS cohort. [file ACN3-9999-0-s001.docx]

**Supplementary Material**

**Table S1: Detailed Alzheimer marker in CSF of FUS-ALS cohort**

|  | **Patient ID** | **Tau**  (pg/ml) | **phosphoTau**  (pg/ml) | **β-Amyloid 1-40**  (pg/ml) | **β-Amyloid 1-42**  (pg/ml) | **β42/β40 Ratio** |
| --- | --- | --- | --- | --- | --- | --- |
| **jALS** | jALS_1 | 281  (ref: 146-410 pg/ml) | 39,2  (ref: < 55 pg/ml) | 5328  (ref: 7231-17404 pg/ml) | 471  (ref: >489.6 pg/ml) | 0.088  (ref: >0.05) |
|  | jALS_2 | N/A | N/A | N/A | N/A | N/A |
|  | jALS_3 | 250  (ref: <290 pg/ml) | 24,3  (ref: < 61 pg/ml) | 6024  (ref: >630 pg/ml) | 1339  (ref: >630 pg/ml) | 0.222  (ref: >0.095) |
| **aALS** | aALS_1 | 103  (ref: <290 pg/ml) | 19,2  (ref: < 61 pg/ml) | 2942  (ref: >630 pg/ml) | 613  (ref: >630 pg/ml) | 0.208  (ref: >0.095) |
|  | aALS_2 | 186  (ref: <290 pg/ml) | 19,7  (ref: < 61 pg/ml) | 4765  (ref: >630 pg/ml) | 1008  (ref: >630 pg/ml) | 0.212  (ref: >0.095) |
|  | aALS_3 | N/A | N/A | N/A | N/A | N/A |
|  | aALS_4 | 255  (ref: 146-410 pg/ml) | 31,5  (ref: 21.5-59.0 pg/ml) | 10623  (ref: 7755-16715 pg/ml) | 1060  (ref: 725-1777 pg/ml) | 1.00  (ref: 0.68-1.15) |
|  | aALS_5 | 695  (ref: <290 pg/ml) | 63.8  (ref: < 61 pg/ml) | 10060  (ref: >630 pg/ml) | 1678  (ref: >630 pg/ml) | 0.167  (ref: >0.095) |

Abbreviations: jALS = juvenile ALS; aALS = adult-onset ALS; ref. = reference; N/A = not assessed.
